# Fever of Unknown Origin in a Patient With Human Immunodeficiency Virus

**DOI:** 10.7759/cureus.49356

**Published:** 2023-11-24

**Authors:** Trishna Kattel, Shreeyanta KC, Jacob Schick, Sze Jia Ng, Oladimeji Lanade

**Affiliations:** 1 Internal Medicine, Crozer-Chester Medical Center, Upland, USA; 2 Radiology, Johns Hopkins University School of Medicine, Baltimore, USA; 3 Internal Medicine, University of Tennessee Health Science Center, Memphis, USA

**Keywords:** anca, vasculitis, granulomatosis with polyangiitis, fever of unknown origin, hiv

## Abstract

Fever of unknown origin (FUO) presents a diagnostic challenge, particularly in patients with human immunodeficiency virus (HIV) due to their immunocompromised state. We report a case of a 21-year-old male with HIV who presented with persistent fever and was found to have a positive proteinase-3 antibody, raising suspicion of granulomatosis with polyangiitis (GPA). The patient's symptoms, negative infectious workup, and elevated proteinase-3 levels prompted consideration of non-infectious etiologies. Despite the absence of renal involvement, corticosteroid therapy was initiated, leading to the resolution of fever. However, the false positive association of proteinase-3 in HIV patients introduces uncertainty regarding the definitive diagnosis of GPA. A tissue biopsy would have provided further clarity, but it was not performed in this case. Our workup aligns more closely with a diagnosis of GPA, considering the patient's response to treatment and the absence of clinical deterioration. This case highlights the complexity of diagnosing non-infectious causes of FUO in HIV-infected individuals. It emphasizes the need for a multidisciplinary approach involving infectious disease specialists and rheumatologists to ensure accurate diagnosis and appropriate management.

## Introduction

A fever of unknown origin (FUO) was initially defined as a persistent fever with a temperature of 38.3 °C (101 °F) or above for at least three weeks, with unknown etiology, despite a minimum of one week of thorough investigations in the hospital [1]. Given the increasing number of patients with immunocompromised status and the availability of complex treatment options, researchers Durack and Street proposed a revised definition and subclassified FUO into classic, nosocomial, neutropenic, and human immunodeficiency virus (HIV)-related FUO [2]. HIV-related FUO poses a diagnostic challenge due to the underlying immunocompromised state and the increased susceptibility to opportunistic infections and malignancies. Non-infectious etiologies, including lymphomas or other inflammatory diseases, should be in consideration in HIV patients with negative infectious workup. However, the diagnosis of non-infectious causes could be complicated by abnormal T cells in these patients. Herein, we reported a unique presentation of FUO in a patient with HIV, who was found to have a positive proteinase-3 antibody, leading to suspicion of granulomatosis with polyangiitis.

## Case presentation

A 21-year-old male with a one-year history of HIV and most recent CD4 of 88, non-compliant with antiretroviral therapy, presented with a two-week history of upper respiratory symptoms, including sore throat, dry cough, and fever. Before his presentation to the hospital, the patient had tested positive for syphilis but did not seek treatment. Upon admission, the patient was diagnosed with oral candidiasis, pneumonia, and syphilis. This was successfully treated with fluconazole, tailored antibiotics, and intramuscular penicillin. He was also restarted on his home highly active antiretroviral therapy (HAART) therapy.

Despite treatment, the patient continued to experience persistent fever as high as 104^o^Fahrenheit. The patient appeared chronically ill but was never in acute distress. No oral ulcers, lymphadenopathy, skin rashes, or retinal changes were noted. His cardiac, pulmonary, gastrointestinal, and genitourinary examinations were unremarkable except for persistent sinus tachycardia. 

Laboratory findings on admission revealed pancytopenia with elevated erythrocyte sedimentation rate (ESR), ferritin, and C-reactive protein (CRP). He also had an AKI, which was resolved with treatment. An extensive infectious workup that included blood cultures, urinalysis and urine cultures, brain magnetic resonance imaging (MRI), computed tomography (CT) of the abdomen and pelvis, lumbar puncture, bone marrow biopsy with cultures, bronchoalveolar lavage, blood cultures, and acid-fast stain all returned negative for an infectious source. This data is provided in Table 1. In light of his persistent fevers of unknown origin (FUO), empiric antifungal coverage with micafungin was added to the current antibiotic regimen, but no improvement was noted. At this point, concern was raised for non-infectious etiology. His proteinase-3 (PR3) immunoassay was found to be elevated at 3.3 Antibody Index (AI) (normal 0-1 AI).

**Table 1:** Diagnostic workup ANA: Antinuclear antibody; CMV: Cytomegalovirus; RPR: rapid plasma reagin; EBV: Epstein-Barr virus; EIA: enzyme immunoassay; RMSF: Rocky Mountain spotted fever; MRSA: methicillin-resistant Staphylococcus aureus; RSV: Respiratory syncytial virus; BAL: Bronchoalveolar lavage; CRP: C-reactive protein; CSF: Cerebrospinal fluid; VDRL: Venereal Disease Research Laboratory

Lab Study	Result
Rheumatoid factor	Negative
ANA	Negative
Proteinase-3 Ab	3.3 AI (<1.0)
Myeloperoxidase Ab	<1.0 AI (<1.0)
Hep A IgM, Hep B Core IgM, Hep Bs Ag, Hep c Ab	Non-reactive
HIV-1 copies/ML	29,97,670
CMV IgM	0.39 (<=0.90)
CMV IgG	5.03 (<=0.90)
Mononucleosis	Negative
Cryptococcal Ag	Negative
RPR Qual	Reactive
RPR Quan	1:32
FTA-ABS	Reactive
Toxoplasmosis IgG Ab	<7.20 IU/mL
TB	0
(1-3)-B-D-Glucan	95 pg/mL (<60)
Parvovirus B19 IgM	0.1 (<0.9)
Parvovirus B19 IgG	2.9 (<0.9)
Babesia microti DNA, RT-PCR	Not detected
EBV IgM Ab	Negative
EBV IgG Ab	501 U/mL (<18.00)
Mycoplasma IgM	89 U/mL
B. henselae IgG screen; titer	Positive; 1:128 (<1:64)
B. henselae IgM screen	Negative
B. quintana IgG screen; titer	Positive; 1:64 (<1:64)
B. quintana IgM screen	Negative
Histoplasma Galact Ag, Ur	<0.2 ng/mL (<0.2)
Aspergillus Ag, EIA, Sr	Not detected
Mycobacterium culture x3	No AFB isolated at 6 weeks
RMSF IgM	Not detected
RMSF IgG	Detected
COVID, Influenza A, B, MRSA, adenovirus, coronavirus, human metapneumovirus, human rhina/enterovirus, parainfluenza, legionella, mycoplasma pneumoniae, Bordetella pertussis, Bordetella parapertussis, Clamydia pneumoniae	Negative
RSV RNA	Positive
BAL	Negative for malignancy, no PCP
CRP	161.1mg/L (<=4.9)
CSF studies	
Glucose	55 mg/dL (50-80)
Protein	25 mg/dL (16-46)
Mononuclear	WBC too low %
Color	Clear
Culture	Negative
WBC	0/uL (0-5)
RBC	21/uL (0-10)
VDRL	Non-reactive
HSV1,2, HHV6, VZV, Cryptococcus neoformans, E coli K1, H. influenza, Listeria monocytogenes, Neiserria meningitidis, Streptococcus agalactiae, Streptococcus pneumoniae, enterovirus	Not detected
sCD25	Negative

**Figure 1:**
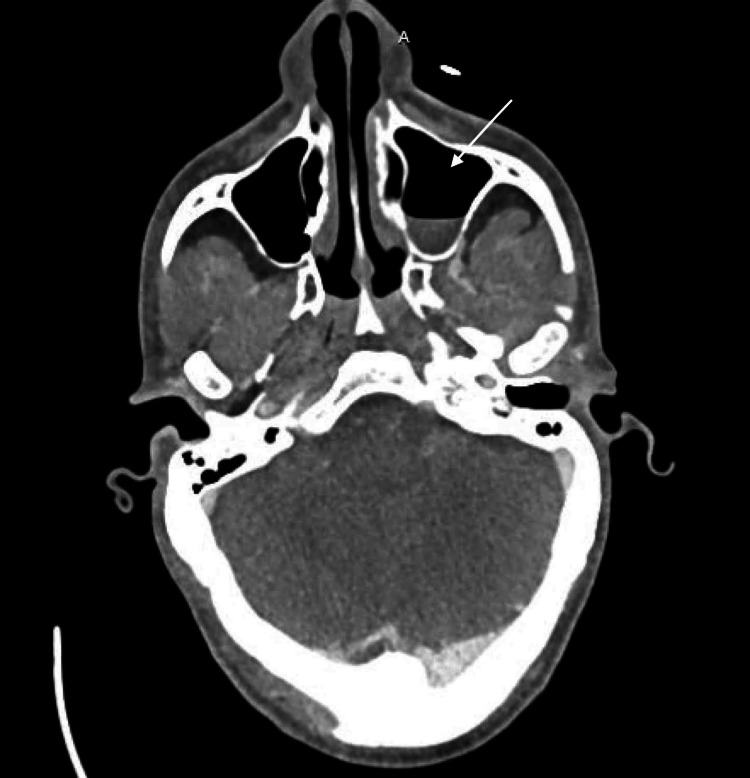
Left Maxillary Sinusitis The arrow points to an air-fluid level of the left maxillary sinus.

This positive value of PR3 antibody level, evidence of sinusitis on imaging (Figure 1), pulmonary symptoms of dry cough and pneumonia, and proteinuria on presentation raised suspicion for granulomatosis with polyangiitis. A trial of corticosteroid therapy was thus initiated, resulting in the resolution of the fever.

## Discussion

The etiologies of fever of unknown origin (FUO) can be broadly categorized as infectious or non-infectious. This challenging diagnosis is further complicated by the presence of HIV in patients. In our case, the patient's negative infectious workup prompted consideration of non-infectious etiologies. Drug fever was initially considered, but discontinuation of antibiotics did not result in the resolution of the fever. Hemophagocytic lymphohistiocytosis (HLH) was also suspected, given his initial pancytopenia and elevated ferritin level. However, his negative CD25 result and improvement of complete blood count throughout the hospitalization make HLH less likely. Lymphoma, a common occurrence in HIV patients, was considered, given his presentation of night sweats, weight loss, and fever. However, imaging showed no adenopathy, and his CD markers for lymphoma returned negative.

A vasculitis workup revealed an elevated proteinase-3 (PR3) antibody level of 3.3 AI. with similar results on the repeat PR3 antibody test. Of note, the patient had evidence of air-fluid levels of the maxillary sinus and proteinuria on admission but no evidence of further respiratory tract involvement or renal dysfunction. According to the 2022 American College of Rheumatology (ACR) diagnostic guidelines for granulomatosis with polyangiitis (GPA) [3], an elevated serum PR3 antibody with any upper respiratory tract involvement meets the diagnostic criteria for GPA. Due to the lack of renal involvement, renal biopsy was deferred. Since there was no convincing evidence of an underlying infectious etiology and the workup suggested the possibility of underlying vasculitis, corticosteroid therapy was initiated. His fever resolved after starting the steroid therapy.

ANCA-associated vasculitis is a relapsing disease characterized by the transient appearance of autoantibodies that trigger an immune response in the vasculature of the kidney's upper and lower respiratory tract. Interestingly, some case reports have described a possibility of false positive association of PR-3 antibodies in HIV patients [4-6]. Studies have correlated the PR3-specific IFNγ responses with the CD4+T cells, characterized by the expression of cytotoxicity-associated molecule GPR56. GPR56, a G-protein coupled receptor in T cells observed in HIV, is linked to cytotoxicity in other lymphocytes, suggesting a possible association with ANCA-associated vasculitis in HIV [7]. While ANCA positivity has been reported in HIV patients, true ANCA vasculitis in these patient is extremely rare [8].

Although our patient met the diagnostic criteria for GPA by the 2022 ACR guideline [3], the false positive association of PR-3 in HIV raises some uncertainty in the diagnosis. Without a tissue diagnosis, we cannot definitively confirm GPA as the etiology of the patient's fever. However, the patient's favorable response to steroid treatment with the absence of clinical deterioration of antibiotics supports a non-infectious etiology of his FUO. Collectively, our workup aligns more closely with a GPA diagnosis.

## Conclusions

Our case highlights the challenges in diagnosing the non-infectious etiology of FUO, particularly in the setting of HIV infection. The persistence of fever despite extensive infectious workup warranted consideration of non-infectious etiologies. The patient's clinical course elevated proteinase-3 levels and response to steroid therapy suggest a probable diagnosis of granulomatosis with polyangiitis (GPA). However, the false positive association of PR-3 in HIV introduces some uncertainty, highlighting the importance of tissue biopsy in these patients. This case emphasizes the need for vigilance in considering non-infectious etiologies and the complexity of diagnosing and managing FUO in HIV-infected individuals. A multidisciplinary approach involving infectious disease specialists and rheumatologists is essential for accurate diagnosis and appropriate management of FUO in HIV-infected patients.
